# Effects of light, electromagnetic fields and water on biological rhythms

**DOI:** 10.1016/j.bj.2024.100824

**Published:** 2024-12-12

**Authors:** Jan Martel, Nicolas Rouleau, Nirosha J. Murugan, Wei-Chun Chin, David M. Ojcius, John D. Young

**Affiliations:** a Center for Molecular and Clinical Immunology, Chang Gung University, Taoyuan, Taiwan; b Department of Health Sciences, Wilfrid Laurier University, Waterloo, Ontario, Canada; c Department of Biomedical Engineering, Tufts University, Medford, MA, USA; d Department of Chemical and Materials Engineering, University of California, Merced, CA, USA; e Department of Biomedical Sciences, Arthur Dugoni School of Dentistry, University of the Pacific, San Francisco, CA, USA; f Chang Gung Biotechnology Corporation, Taipei, Taiwan

**Keywords:** Chronobiology, Electromagnetic fields, Light therapy, Metabolic disorders

## Abstract

The circadian rhythm controls a wide range of functions in the human body and is required for optimal health. Disruption of the circadian rhythm can produce inflammation and initiate or aggravate chronic diseases. The modern lifestyle involves long indoor hours under artificial lighting conditions as well as eating, working, and sleeping at irregular times, which can disrupt the circadian rhythm and lead to poor health outcomes. Seasonal solar variations, the sunspot cycle and anthropogenic electromagnetic fields can also influence biological rhythms. The possible mechanisms underlying these effects are discussed, which include photoentrainment, resonance, radical-pair formation, ion cyclotron resonance, and interference, ultimately leading to variations in melatonin and cortisol. Intracellular water, which represents a coherent, ordered phase that is sensitive to infrared light and electromagnetic fields, may also respond to solar variations and man-made electromagnetic fields. We describe here various factors and underlying mechanisms that affect the regulation of biological rhythms, with the aim of providing practical measures to improve human health.

## 1 Introduction

Biological rhythms involve changes in living organisms that vary as a function of daylight, seasons, and the solar cycle. The most studied biological rhythm is the 24-h circadian clock which is observed in most living organisms [1]. Light from the Sun is the main Zeitgeber that entrains the circadian rhythm and sleep/wake cycle to a period of 24 h, corresponding to one full rotation of the Earth on its axis relative to the Sun [1]. Under controlled light and temperature conditions, the circadian rhythm persists but its period becomes shorter or longer than 24 h [2]. The circadian rhythm plays an important role in maintaining the regularity of daily body functions and ensures their synchronization with environmental changes-especially those of the Sun. Moreover, the circadian rhythm is thought to provide advantages for living organisms by synchronizing body functions according to time of the day and favoring optimal use of the limited resources and energy available. Indeed, extrinsic controls of nutrient consumption, ambulation, sexual behavior, and rest or sleep are likely to increase fitness outcomes relative to ad hoc decision-making by the organism.

The circadian clock controls most physiological functions including sleep/wake, fasting/eating, and anabolic/catabolic cycles, as well as variations in body temperature, hormones, and immune functions [3]. Disruption of the circadian rhythm by sleep deprivation, night shift work, and jet lag is associated with sub-optimal body functions, inflammation, and various symptoms (e.g., fatigue, fever, discomfort, pain, headache). Given the importance of the circadian rhythm, it is not surprising that chronic disruption is associated with the development of some of the most prevalent diseases and causes of death such as type 2 diabetes, obesity, cardiovascular disease, cancer, mood disorders, and neurological diseases [4]. A bidirectional relationship exists between the circadian rhythm and disease in which circadian disruption increases disease severity while many disease-causing factors such as stress and substance use can disrupt circadian rhythmicity [4].

**Fig. 1:**
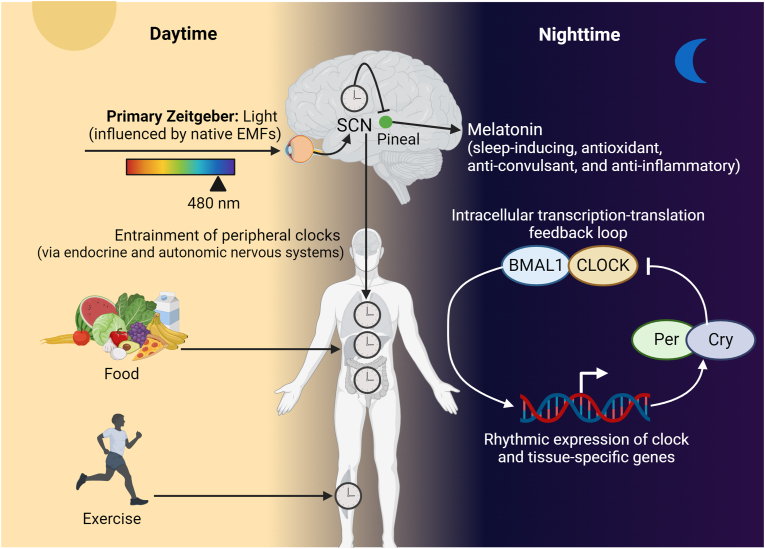
**Regulation of the human circadian rhythm.** Light entrains the circadian clock in the suprachiasmatic nucleus (SCN) which controls hormone production by the hypothalamus and peripheral clocks in various organs via the autonomous nervous system. Light also inhibits melatonin production by the pineal gland. Food and exercise influence circadian clocks in peripheral organs. The Earth's electromagnetic fields (EMFs), including the geomagnetic field and Schumann resonances, may also influence the circadian rhythm, either directly or indirectly. Abbreviations: Cry: cryptochrome; Per: period. The images shown in this article were created using BioRender.

In order to maintain circadian rhythmicity, the suprachiasmatic nucleus (SCN) of the hypothalamus must be reset by daily light that activates photoreceptors in the retina [Fig. 1]. Neuronal signals from this “master clock” are then relayed to several peripheral clocks that regulate the function of other organs such as the heart, liver, and lungs via the autonomic nervous system and hormones such as glucocorticoids, catecholamines, and melatonin. The circadian rhythm involves intracellular transcription-translation feedback loops in which rhythmic expression of clock genes regulate the expression of downstream gene products, followed by repression of their own expression [Fig. 1]. In most vertebrates, the transcription factors CLOCK and BMAL1 modulate the expression of period (*Per1, 2, 3*) and cryptochrome (*Cry1, 2*) genes, which in turn repress gene expression to allow a new cycle [5]. This oscillating molecular clock regulates thousands of genes in a tissue-specific manner [Fig. 1].

Efforts to study biological rhythms have contributed to a deeper understanding of behavioral patterns and their associated health outcomes. The founder of chronobiology, Franz Halberg, observed in the 1950s that completely opposite biological responses could be produced depending on when laboratory measurements were made [6]. For instance, the level of eosinophils was reduced in calorie-restricted animals when sampled in the evening, but no variation or even the opposite response was observed when measurements were made in the early morning [6]. It was later shown that human immune functions peaked during the night and are suppressed by cortisol during the day, whereas sympathetic tone increased in the early morning hours, which coincided with the most likely period for heart attacks within a 24-h period (blood pressure peaks at 9 a.m.) [6]. These differences between morning and evening measurements were enhanced by caloric restriction [6]. Furthermore, variations in the cyclic fluctuations of immune cells occurred between mouse strains due to genetic make-up. Although many advances have been made, Halberg's conclusion that the spot-checks and time-unqualified average values measured in medicine (and biological research) should be transformed into time-series observations and inter-individual assessments is still relevant today.

## 2 Light and the circadian rhythm

Independently of phototransduction within rods and cones of the retina, intrinsically photosensitive retinal ganglion cells (ipRGCs) expressing the non-visual melanopsin (OPN4) respond to blue light with a peak of sensitivity at wavelengths of 480 nm. In parallel with visual pathways, this signal inhibits melatonin production by the pineal gland and activates hormone secretion by the hypothalamus [Fig. 1]. Notably, red wavelengths (595–660 nm) do not inhibit melatonin production [7], which highlights the specificity of the response. Several other non-visual opsins, including encephalopsin (OPN3), are widely distributed throughout the central nervous system and respond to narrow bands of blue light (OPN3: 463 nm) [8]. Indeed, there are many photoactive molecules within the brain, including naturally fluorescent amino acids such as tryptophan, neurotransmitters like serotonin, as well as highly conserved flavins and porphyrins [9,10]. Together, these molecules may serve as activators and amplifiers of direct neural transduction of light toward the maintenance of circadian rhythmicity.

Intense light during daytime is associated with an improved circadian rhythm and physiological functions. For instance, diurnal sand rats exposed to full-spectrum light at 3000 lux in the morning showed high-amplitude circadian rhythms, improved glucose homeostasis, and reduced body weight, as well as reduced anxiety- and depression-like behaviors compared to controls [11]. In humans, a crossover, randomized clinical trial of 34 overweight women indicated that a 45-min bright light treatment in the morning for 3 weeks reduced body fat and appetite [12]. Bright light therapy in the morning also improved the circadian rhythm and was used to treat seasonal affective disorder and winter depression by reducing neuro-inflammation and improving sleep quality [13].

In contrast, light at night disrupts the circadian rhythm and is associated with inflammation, obesity, and the development of chronic diseases [2,4]. This is attributed partially to a reduction of melatonin, which is not only a sleep-inducing, antioxidant, anticonvulsant, and anti-inflammatory molecule, but also a hormone involved in body repair during sleep and key control of hormonal and immune activities throughout the body. In mice, illumination during the dark phase increases body weight and impairs glucose tolerance without affecting daily calorie intake or activity [14]. A prospective study conducted on 678 elderly participants with no evidence of diabetes at baseline showed that light at night (>5 lux) was associated with increased incidence of type 2 diabetes [15]. Similarly, a cross-sectional analysis of over 113,000 women showed that light exposure at night was associated with high body mass index (BMI) and obesity [16]. Consistent with these studies, shift work is associated with increased incidence of metabolic syndrome, diabetes, and obesity [17]. A meta-analysis of 17 studies indicated that light exposure at night was associated with a dose-dependent increase of breast cancer, especially in pre-menopausal women [18].

The increased use of blue-enriched lights, television, computer monitors, smartphones, and tablets throughout the day has become a major disruptor of circadian rhythmicity in the general population. Moreover, the recent change of incandescent lightbulbs for light-emitting diodes (LEDs) is also associated with detrimental effects on health due to their increased white and blue wavelengths and reduced infrared light. These effects are due at least in part to the observation that infrared light is beneficial for the body in various ways [9]. For instance, infrared light induces ATP production by mitochondria, reduces pain and inflammation, and improves recovery and healing [9]. Interestingly, action potentials from neurons can be blocked by infrared light [19], suggesting that infrared wavelengths may decrease the excitability of neural tissues, which in turn can reduce pain and induce relaxation and body repair [9]. However, adjacent wavelengths may produce opposite effects by alternative pathways, such as increased cortical excitability [20]. Office workers show a higher incidence of skin melanoma compared to outdoor workers, possibly due in part to the effects of fluorescent, LED and compact fluorescent lights, which produce high-frequency voltage transients (i.e., dirty electricity), radiofrequencies, and a high body voltage [21].

Solutions to counter circadian disruption related to light are available and include more exposure to sunlight in the early morning and during the day, phasing-out fluorescent lights and energy-saving, blue-enriched LEDs, reduction of the use of smartphones, tablets, and computers in the evening and at night, and a change to more red wavelengths and indoor light intensities that more closely simulate the intensity and spectral properties of the Sun.

## 3 Nutrition, exercise, aging, and the circadian rhythm

The circadian rhythm can also be influenced by behavioral cues such as eating and exercise. Eating most calories at lunch time and avoiding large evening dinners and night snacks favors digestion and metabolic functions that are aligned with the circadian rhythm and daytime digestive and metabolic activities [22]. For instance, controlled clinical trials in which people followed early time-restricted eating aligned with the circadian rhythm was associated with improvements in insulin sensitivity, blood pressure, and immune functions [23,24]. Exercise is also a potent Zeitgeber for skeletal muscle clocks which may be used to offset the effects of disrupted sleep cycles. It has been suggested that exercising in the afternoon and early evening to synchronize muscle functions with the circadian rhythm may enhance the beneficial effects of exercise on hormones and metabolism and reduce metabolic diseases [25].

**Fig. 2:**
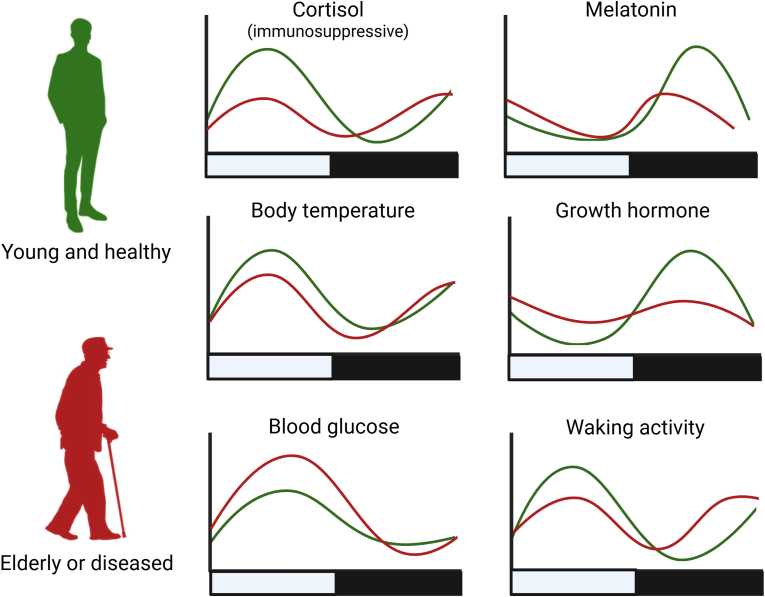
**Differences in circadian rhythms between young and healthy adults versus elderly or diseased individuals.** Amplitudes and phases of hormones and physiological functions vary between young and healthy adults (green curves) and elderly or diseased individuals (red curves). Modified and adapted from the work of Hood and Amir [26] which is under a Creative Commons license.

Aging has been shown to alter the circadian rhythm in various ways. Older adults show altered circadian rhythms characterized by a morning chronotype and altered rhythmicity of various physiological functions [Fig. 2] [26]. For instance, advanced phase and reduced amplitude in the production of cortisol and melatonin have been observed in older individuals [Fig. 2] [26]. Accumulation of senescent cells and the reduced production of cortisol—an immunosuppressive hormone produced by adrenal glands in the morning—are involved in the development of *inflammaging*, a condition of low, chronic inflammation observed in aging and many elderly people [27]. Inflammation that increases with aging can therefore disrupt the circadian rhythm; conversely, disruption of the circadian rhythm can produce inflammation, possibly producing a persistent positive feedback loop. Besides inflammation, variations involving yellowing and thickening of the eye lens may reduce sensitivity to light during aging, whereas neurodegeneration and pineal calcification may affect SCN firing [26] and melatonin production [28], respectively.

One of the factors involved in aging and the development of neurodegenerative diseases is low vitamin D production, which is common in the population and essentially reflects an indoor lifestyle and inadequate sunlight exposure. However, dementia-free older adults who took an oral vitamin D_3_ supplement for at least 146 days per year had a 1.8-fold increased risk of dementia compared to people not taking the supplement [29]. In people with pre-dementia, continuous vitamin D_3_ intake was associated with a 2.2-fold higher risk of mortality. In the same study, supplementation with vitamin D_3_ increased amyloid-beta deposition in the brain and exacerbated Alzheimer's disease in mice [29]. It thus appears that oral vitamin D supplementation does not offset the detrimental effects of lack of sunlight and abnormal blood vitamin D levels on brain function.

Various lifestyle interventions such as regular exercise, intermittent fasting, fasting-mimicking diets, and caloric restriction mimetics are known to produce health benefits and anti-aging effects, including induction of autophagy, rejuvenation of immune function, and enhanced resistance to stress [30]. Increased sunlight exposure, adequate sleep, and stress management may also be added to these healthy interventions.

While several advances have been made in our understanding of the importance of the circadian rhythm, many unexplained phenomena remain. For instance, it has been shown that red blood cells which lack a nucleus (and therefore transcription-translation feedback loops which are believed to maintain endogenous circadian rhythms) show circadian rhythmicity in reactive oxygen species (ROS) when analyzed ex vivo [31]. Moreover, removal of the SCN in mice dampens the amplitude of variables that follow a circadian rhythm, but does not completely abrogate their circadian rhythmicity [6]. The mechanism underlying the regulation of biological rhythms based on seasons and the solar cycle also remains unclear. These observations indicate that other factors influence circadian and biological rhythms. Because many cells display non-associative learning response [32], the possibility that some biological rhythms are entrained by extrinsic and adjacent clocks deserves further attention.

## 4 Electromagnetism: an overlooked factor

In addition to light and diet, the circadian rhythm is also influenced by electromagnetic fields, as shown in various living organisms including insects, rodents, and humans [33,34]. The Earth's electromagnetic fields comprise the geomagnetic field, Schumann resonances, and the global electrical circuit [28,34]. The geomagnetic field is created by Earth's rotation and electric currents in the molten iron core, producing a static magnetic field with average intensity of 35 μT near the equator and 60 μT at latitudes closer to the north and south poles [28]. Schumann resonances are electromagnetic waves of low intensity that reverberate between the ionosphere and the Earth's surface. Schumann resonances are the result of continuous global lightning discharges that produce a fundamental frequency of 7.8 Hz due to the circumference of the Earth, along with harmonics of 14.1, 20.3, 26.4, and 32.5 Hz. Their intensity is particularly low, with an electric component of 10 mV/m and a magnetic component of 1–10 nT [28].

While the global electrical circuit is influenced by weather, both the geomagnetic field and Schumann resonances show a constant diurnal variation in intensity that follows sunlight intensity (i.e., peaking at noon and reaching lowest levels at night), in a manner highly similar to the circadian rhythmicity of body functions [35,36] [Fig. 2]. This observation led many researchers to suggest that natural electromagnetic fields may also be involved in entrainment of the circadian rhythm [[34], [35], [36]].

Rütger A. Wever observed that healthy volunteers maintained for weeks in a bunker shielded from natural electromagnetic fields showed desynchronized circadian rhythms characterized by longer phase periods and changes in body temperature and activity [33]. Notably, it was possible to restore the volunteers' circadian rhythms using an artificial electrical field that was activated during the day, oscillating at an extremely low frequency (ELF; 10 Hz) [33]. Experiments in which animals were shielded from the Earth's geomagnetic field also showed disrupted circadian rhythms that contributed to the development of sleep disorders and metabolic and neurological diseases [37]. Humans shielded from environmental magnetic fields displayed reduced theta-band (4–7 Hz) spectral power when measured with electroencephalography (EEG) [38], whereas exposure to artificially-generated geomagnetic field conditions in a controlled laboratory setting modulated alpha rhythms (8–13 Hz) in real-time [39]. Notably, both theta and alpha rhythms during waking and sleep states are modulated by administrations of melatonin [40]. Indeed, mortality in rat models of acute limbic lability (with epileptic seizures) was found to be significantly correlated with geomagnetic activity in the days immediately preceding death, and this was linked to indicators of nocturnal melatonin suppression [41].

The incidence and mortality of many chronic diseases including cardiovascular disease, depression and Covid-19 have been shown to increase following solar storms, which affect the Earth's electromagnetic fields [28,42]. Cherry proposed that Sun-induced variations in Schumann resonances may be responsible for the detrimental effects of solar and geomagnetic storms on human health [35]. More recently, Krylov and colleagues showed that simulated geomagnetic storms affect circadian rhythms in model organisms (i.e., cockroaches and snails) [43]. Moreover, the same group showed that simulations in which the natural geomagnetic field was shielded and replaced by an artificial magnetic field could be used to entrain the circadian rhythm of zebrafish to the unusual long period of 36 h [44]. Notably, the quasi-static magnetic field used in this study slowly increased in intensity in a manner similar to the diurnal variation of the geomagnetic field [44]. Another study showed that an ELF magnetic field (0.1 mT, 50 Hz) induced the cyclic expression of circadian rhythm genes (i.e., *BMAL1*, *PER2*, *PER3*, *CRY1*, *CRY2*) in human cultured fibroblasts [45].

Charged particles from the solar wind that become trapped into the Van Allen belts are sensitive to the gravitational force from the Moon, producing the lunar variation in the geomagnetic field, which can also affect living organisms. Frank A. Brown Jr. observed that fiddler crabs and oysters maintained their monthly locomotor activity and valve opening in relation to the lunar cycle, even when kept in darkness or dim light [46]. The lunar cycle was also found to influence human sleep, with a full Moon being associated with reduced deep sleep, sleep quality, and melatonin levels [47]. The lunar phase influences not only plants and animals but also humans, as seen from the increase of epileptic seizures, violent behaviors and spontaneous abortions observed during full and/or new Moon (see the excellent review in Ref. [48]).

## 5 What is the mechanism?

It has been proposed that, under some conditions, resonance occurs transiently between Schumann resonances and brain waves [35], as well as between the geomagnetic field and the heart's magnetic field [49]. Such resonance may provide information about the time of the day which in turn entrains circadian rhythmicity in biological functions. In nature, oscillatory systems tend to synchronize their activities using chemical, electrical, mechanical and gravitational interactions, even if their frequencies are different initially [50]. While the effects of light and natural electromagnetic fields overlap, the geomagnetic field may represent the only Zeitgeber in environments with constant temperature and illumination such as caves and deep sea [44].

**Fig. 3:**
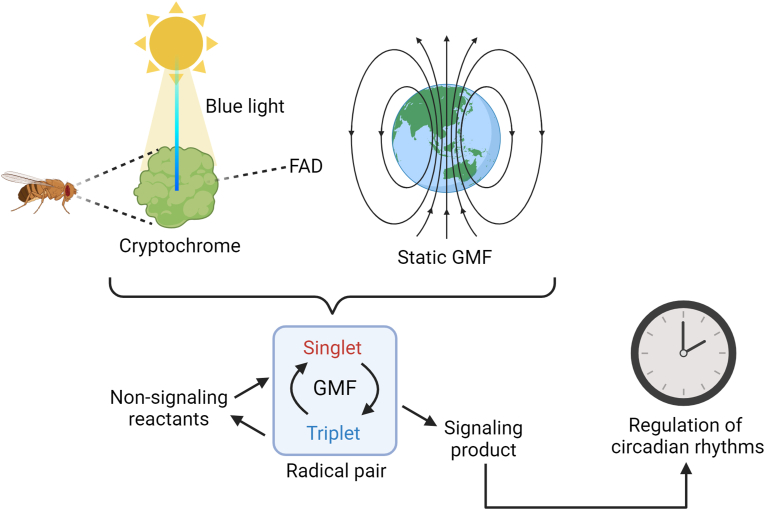
**Radical pair mechanism involved in circadian control in some organisms.** Abbreviations: GMF, geomagnetic field; FAD, flavine adenine dinucleotide. Modified and adapted from the work of Abeyratne et al. [52].

Melanopsin-deficient mice showed reduced photoentrainment [51], suggesting the involvement of other photoreceptors. In *Drosophila*, cryptochromes regulate the circadian rhythm via the radical pair mechanism [52] which shares some similarities with the magnetoreception-based compass used by migratory birds [53]. The observations that cryptochromes are conserved in mammals and are sensitive to weak magnetic fields suggest a possible involvement in the control of the circadian rhythm in mammals as well [53]. In this context, the 25–50 nT variation in the intensity of the geomagnetic field that occurs daily [36] may induce changes in light-induced production of free radicals within the retina, leading to downstream effects on the circadian clock [52]. The proposed mechanism involves light-induced formation of radical pairs in retinal flavin co-factors associated with cryptochromes, with the spin state of free radicals being sensitive to weak magnetic fields [52,53] [Fig. 3]. More studies are needed to determine whether a similar mechanism is involved in circadian rhythm control in animals and humans.

Observations by Liboff indicate that the daily increase of 25–50 nT in the intensity of the geomagnetic field may regulate and influence the circadian rhythm [28,36]. This is based on the observation that birds that use the geomagnetic field as a compass during migration are also sensitive to similar small variations in magnetic fields [52,53]. As mentioned above, it was possible to entrain the circadian rhythm in zebrafish by simulating this slow increase in static magnetic field intensity [44]. Furthermore, many studies have shown that living organisms react to small variations in magnetic field within the context of the ion cyclotron resonance (ICR) mechanism [28,36,54]. The ICR mechanism is based on the observation that a combination of static magnetic field and time-varying or oscillating magnetic or electric field (oriented parallel or perpendicular, respectively, to the static magnetic field) can produce biological effects on a wide range of cells and living organisms, in spite of their extremely low intensity (as low as 40 nT) [54]. According to Liboff, the static magnetic field represents the Earth's geomagnetic field which enters in resonance with endogenous, oscillating electric or magnetic fields produced by ions and charged molecules in the human body [54]. If were the case, then slight variations in the intensity of the geomagnetic field—as it occurs during solar storms or seasons—may alter resonance in living organisms and affect not only the circadian rhythm but also a wide range of cellular functions. Accordingly, coupling between the geomagnetic field and biochemical reactions has been observed in several cases. For instance, inversion of the horizontal component of the Earth's magnetic field was shown to reduce pineal serotonin-N-acetyltransferase activity and melatonin production in rodents [55].

The biological relevance of the ICR mechanism is shown by the observation that magnetic fields matching ICR frequencies for biologically-relevant ions can influence biological processes in a wide range of living organisms, including in diatom movement, plant growth, bone healing, stem cell differentiation, and animal behaviors, among others [28,54]. When the ratio of the frequency of the time-varying or oscillating magnetic or electric field divided by the intensity of the static magnetic field equals the charge-to-mass ratio of a biologically relevant cation, such as calcium, potassium, magnesium or positively-charged amino acids, the magnetic field treatment produces the same biological effects than if the living organism were treated with the said cation. For instance, magnetic field treatments matching ICR frequency ratio for calcium activate cells, whereas ICR frequencies for potassium inhibit the same reaction [28]. Furthermore, ELF electric and magnetic fields present in the environment that match the ICR frequencies of specific ions can combine with the geomagnetic field to affect biochemical reactions in vivo, providing a possible mechanism for the detrimental effects of power lines and non-ionizing electromagnetic radiation that is modulated at ELF.

Common objections to the possibility that the geomagnetic field may influence physiological functions are usually related to the low signal-to-noise ratio and the low intensity of the field in comparison to fields produced by everyday electric apparels. However, William R. Adey showed that biological organisms are sensitive to weak electromagnetic fields within small windows of frequencies and intensities in spite of thermal noise [28], whereas electromagnetic fields of higher intensities may not necessarily produce interference. Some organs in the body such as glands and sensory organs may be particularly sensitive to the weak change in the geomagnetic field. For instance, variations in the intensity or orientation of ambient magnetic field can inhibit melatonin production by the pineal gland and retina, indicating that these organs are sensitive to the geomagnetic field [56,57]. The presence of paramagnetic magnetite nanoparticles may also modulate or amplify the effects of the geomagnetic field within specific organs such as the brain and endocrinal glands [34].

## 6 Biological rhythms related to seasons and the solar cycle

Cases of influenza increase every winter in the northern and southern hemispheres [58]. In the United States, the incidence and mortality of various chronic diseases increase during the winter, including cardiovascular, respiratory, neurodegenerative, cancer, and kidney disease [59]. Pro-inflammatory cytokines spontaneously increase in humans during the winter in northern and southern hemispheres [60], indicating that winter may induce inflammation and symptoms in a periodic manner, independently of pathogens. Melatonin increases in the winter when nights are longer, whereas it decreases in the summer when nights are shorter, a process that affects reproduction patterns in animals [61]. Moreover, darkness may reduce cortisol level and increase inflammation [62]. Seasonal variations in melatonin and cortisol may thus be involved in the increased incidence and severity of chronic diseases in the winter. Various investigators observed that temperature and climate can affect human health in a seasonal manner, but only around 30% of the variance in the physiological and pathological processes examined could be explained by weather [63]. In addition to many other contributing factors such as aging and existing chronic diseases, it appears likely that variations in sunlight and possibly the seasonal weakening of the Earth's electromagnetic fields may also contribute to disease seasonality [34].

The possibility that the solar cycle may somehow affect human behavior has intrigued many researchers for a long time. Alexander Chizhevsky was among the first researchers to find evidence that sunspot maxima are associated with variation in crop yield and upticks of epidemic diseases and cardiovascular mortality [64]. More recent observations have confirmed that human epidemics and pandemics usually occurred during periods of sunspot minima or maxima [65]. Accordingly, the Covid-19 pandemic started at the end of 2019 during a period of sunspot minimum [65] involving a large north/south asymmetry on the sun [66], and the pandemic lasted roughly 3 years, consistent with potential beneficial effects of a return towards middle sunspot activity [66]. A similar mechanism based on melatonin variation with the solar cycle may explain variation in disease severity with the sunspot cycle. Accordingly, Bartsch and colleagues showed that melatonin levels in rats maintained in constant light-dark laboratory conditions vary based on the sunspot cycle and seasons, but these trends may be disrupted by geomagnetic activity and other environmental factors [67].

Peaks of Covid-19 incidence and mortality in the northern hemisphere occurred not only every winter [42] (consistent with a reduction of infrared light which is anti-inflammatory [9] and with the seasonal weakening of the geomagnetic field as proposed earlier [34]), but also during prolonged periods of geomagnetic disturbances [42]. Moreover, Covid-19 incidence and mortality was higher with increasing latitudes away from the Equator [68], consistent with the notion that disturbances of the Earth's electromagnetic fields affect human health more at high latitudes in the northern and southern hemispheres [69]. While countries at higher latitudes experience colder and drier conditions that are believed to favor viral spread, variations in sunlight, weather and natural electromagnetic fields that affect the host may also be involved in this process.

Through years of meticulous experiments, Giorgio Piccardi noticed that the kinetics of chemical reactions in vitro followed seasonal solar activity and the sunspot cycle [70]. Chemical reactions involving the precipitation of oxychloride of bismuth and blood coagulation deviated from their normal rate based on sunspot number and correlated with daily measurements of atmospheric electric potentials [70]. Moreover, a grounded shield of copper acting as a Faraday cage attenuated these environmental effects and slowed down the chemical reactions compared to unshielded conditions. Capel-Boute later confirmed these observations and noted that chemical reactions also showed a diurnal variation similar to the circadian rhythm and daily solar activity [71]. Piccardi attributed these effects to ELF electromagnetic fields from the Sun and their possible effects on water. If the kinetics of chemical reactions on Earth are robustly affected by solar activity, and human health is dependent on the regularities of electrochemical reactions in cells and tissues throughout the body, it is important to consider the role of space weather in public health.

Circadian oscillations of chemical reactions in aqueous media have also been observed by Voeikov and colleagues [72]. Light emission was monitored from a solution of bicarbonate ions that was enhanced by luminol. These experiments were conducted under controlled light and temperature conditions in order to prevent any effect of these factors [72]. Oscillations in the rate of chemical precipitation, polymer formation, and phase shift in the freezing of supercooled water have been observed to follow daily sun intensity and sunspots [70,71]. In addition to the melatonin-based mechanism suggested above, the seasonal and sunspot-related variations in the Earth's electromagnetic fields may influence biological rhythms in living organisms, possibly through some effects on water.

## 7 A possible role for liquid crystalline water

**Fig. 4:**
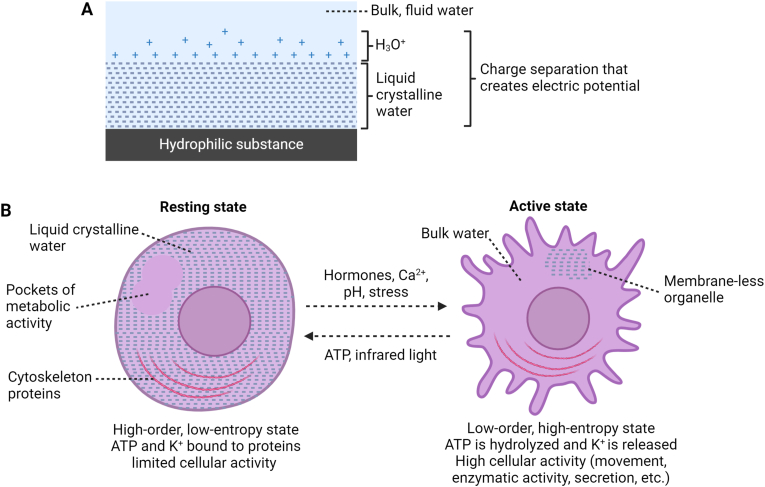
**Liquid crystalline water and cellular activation.** (A) Hydrophilic substances such as cell membranes and proteins induce the formation of negatively-charged liquid crystalline water, also called exclusion zone (EZ) water, creating an electric potential. (B) Resting cells may contain high levels of EZ water, whereas activated cells contain fluid water that allows movement, contraction, and other cellular activities depending on cell organization. Cells alternate between resting and activated states following a water phase shift produced by the factors indicated. Abbreviation: ATP: adenosine triphosphate.

More than 130 years ago, the discoverer of X-ray, Wilhelm C. Röntgen, suggested that water may be found in two phases, a liquid and disordered form, such as the phase people are familiar with, and a crystalline, gel-like form similar to ice [73]. Albert Szent-Györgyi and Gilbert Ling later proposed that layers of ordered water may surround biological substances such as cell membranes and proteins [63] (Fig. 4). Ling's association-induction hypothesis described the cell cytoplasm as an ion-exchanged resin, that spontaneously binds potassium and excludes sodium, interspersed with this ordered water phase [63]. In the resting state, adsorption of adenosine triphosphate (ATP) to intracellular proteins leads to binding of potassium and water structuring, which together produces a state of low entropy and high potential energy. Stimulatory signals that activate cells such as hormones in turn lead to ATP hydrolysis and liberation of the energy stored in the low entropy state through water destructuring and desorption of bound ions (Fig. 4).

Giuliano Preparata and Emilio Del Giudice used a quantum electrodynamics approach to predict the physical behavior of water [74,75]. Their model showed that water can form two phases: a disordered phase of fluid, bulk water interspersed with clusters of water molecules that vibrate in unison with a trapped electromagnetic field. At a temperature of 30 °C, it was estimated that the ordered phase consisting of coherent domains (CDs) with a diameter of 100 nm represents 40% of water, whereas the bulk phase comprises 60% [76]. CDs were proposed to represent a source of quasi-free electrons for redox reactions inside cells and may absorb energy from the environment and release it to activate biochemical and enzymatic reactions [76]. In addition, due to their quantum coherence and high sensitivity to external energy, it was proposed that CDs may represent the entities that are sensitive to weak ELF electromagnetic fields from the environment [77].

Gerald Pollack and colleagues showed that an ordered water phase forms on hydrophilic substances such as Nafion polymers, muscles, and cell membranes [78]. This ordered crystalline water phase was shown to be more viscous than bulk water, to be negatively charged compared to the bulk water at the interface which accumulates protons, to form layers on hydrophilic substances that can expand to hundreds of micrometers, and to exclude most solutes such as sodium and organic molecules [78]. This exclusion zone (EZ) water is ordered and coherent (i.e., liquid crystalline), therefore showing similarities with the model proposed by Preparata and Del Giudice. It was deduced that hydrophilic substances can split water molecules, producing negative moieties that form EZ layers and protons in the form of hydronium ions (H_3_O^+^) [28]. Therefore, the formation of EZ water creates an electric potential that can be used to produce mechanical or chemical work [78] [Fig. 4]. Liquid crystalline water or EZ may also release electrons which can be used in redox reactions [[75], [76], [77]].

A possible link between water and the circadian rhythm comes from the observation that EZ water is sensitive to changes in light and electromagnetic fields. Accordingly, the formation of EZ water on hydrophilic surfaces can be enhanced by infrared light [28,78]. Moreover, a weak electric field of 7.8 Hz corresponding to the primary Schumann resonance mode can enhance the formation of EZ water on hydrophilic surfaces [79]. In this case, the electric field was only effective when the electrodes were installed above the chamber, indicating the importance of directionality. EZ water also formed at both the north and south poles of a permanent magnet [80]. Therefore, the formation of EZ in living organisms may be sensitive to variations in solar activity and electromagnetic fields.

EZ water is involved in various cellular and physiological processes, including cell division, movement, secretion, and transport [81]. Notably, EZ water can maintain the electrical potential of the cell, without the need for an intact lipid membrane or sodium/potassium pumps [81]. Many cell types are activated by stimuli such as calcium influx that induce a phase shift between the resting gel phase and the activated bulk water phase [Fig. 4]. Moreover, recent experiments indicate that EZ water that forms on the internal surface of blood vessels contribute to blood flow [82].

Given that ICR-like reactions require water [54], it appears that the magnetic field at the ICR frequencies may induce cellular effects by reproducing the electromagnetically-based informational signal of ions, which then affects water and cellular activity. This is consistent with the proposition that electromagnetic signals that are specific for particular ions and molecules may mimic the effects of these substances [83]. Using a weak oscillating magnetic field corresponding to the ICR frequency for H_3_O^+^, it was possible to increase the formation of EZ water and reduce pH [84]. Together, these observations indicate that ELF electromagnetic fields such as the static geomagnetic field may affect water and be involved not only in biological rhythms but also in cellular reactions.

The circadian rhythmicity in the levels of ROS in human cells as mentioned earlier may be due to a diurnal variations in weak environmental electromagnetic fields that in turn influence water via the ICR mechanism or the production of ROS [72]. Voeikov and colleagues observed spontaneous circadian rhythms of photon emission in bicarbonate solutions in which H_2_O_2_ and luminol were added at time zero [72]. Similar cyclic chemical reactions involving ROS and antioxidants have been observed in living organisms [31], but whether they control the cell's circadian rhythmicity remains to be investigated.

Water represents 70% of the human body by weight and 99% by the number of molecules. It contains many highly-mobile charged molecules in the form of protons which are involved in the formation and phase transition of EZ water. Several factors suggest that water (and living organisms which are systems far away from thermodynamic equilibrium) may be particularly sensitive to weak external electromagnetic fields such as the geomagnetic field. It is now evident that water continually absorbs energy from the environment which may influence CDs and EZ water [76]. Both an excess and deficiency of this external energy may be detrimental to human health due to altered kinetics of biochemical reactions occurring in the human body. Intermediate levels of sunspots and seasonal solar energy may provide optimal levels for physiological functions, whereas maxima and minima produce detrimental effects on health. Accordingly, both maximum and minimum solar activity have been shown to produce negative effects on human health [34], [66], [69], [83].

During periods of sunspot minima and maxima, or during winter, a small variation of energy can destabilize the system [83]. For instance, research has shown that weak electromagnetic fields of a few hundred nT reduce melatonin in the winter in animals, whereas an increase of melatonin is observed during the summer [85]. The increase of melatonin production in the summer in this context may be related to a compensatory response aimed at maintaining homeostasis, consistent with the process of hormesis.

While these subtle solar phenomena likely affect all life forms, they may produce observable symptoms of rhythm disruption only in a fraction of individuals at any time. For instance, it was estimated that solar storms affect 10–15% of individuals (i.e., mainly old and diseased) and that people at high and low latitudes closer to the poles are more affected [69]. Indeed, psychiatric hospital admissions, epileptic seizures, and mental health events are positively correlated with geomagnetic storms [86,87]. Healthy individuals react to stress from solar and geomagnetic disturbances by inducing compensatory physiological reactions in order to maintain homeostasis [88]. The health condition of the host will therefore determine whether effects are observed, with the elderly and diseased individuals and those with a high burden of stress and instability being more likely to show symptoms as their physiological functions already show some disorders. Living organisms can adapt to various forms of stress, including electromagnetic stress, but they require time to adapt to a new baseline. Similarly, people who show symptoms of rhythm disruption and jet lag adapt to travel across several time zones and recover within a few days.

## 8 Coherence and synchronization of physiological functions

In addition to CDs and EZ water, the concept of coherence has been used to describe the coupling and synchronization between oscillating systems [63]. Various researchers proposed that the human body may consist of multiple molecular oscillators that are interlocked and entrained to the environment [63]. We have seen that light, the geomagnetic field, and Schumann resonances may act as environmental oscillators that entrain these biological rhythms.

**Fig. 5:**
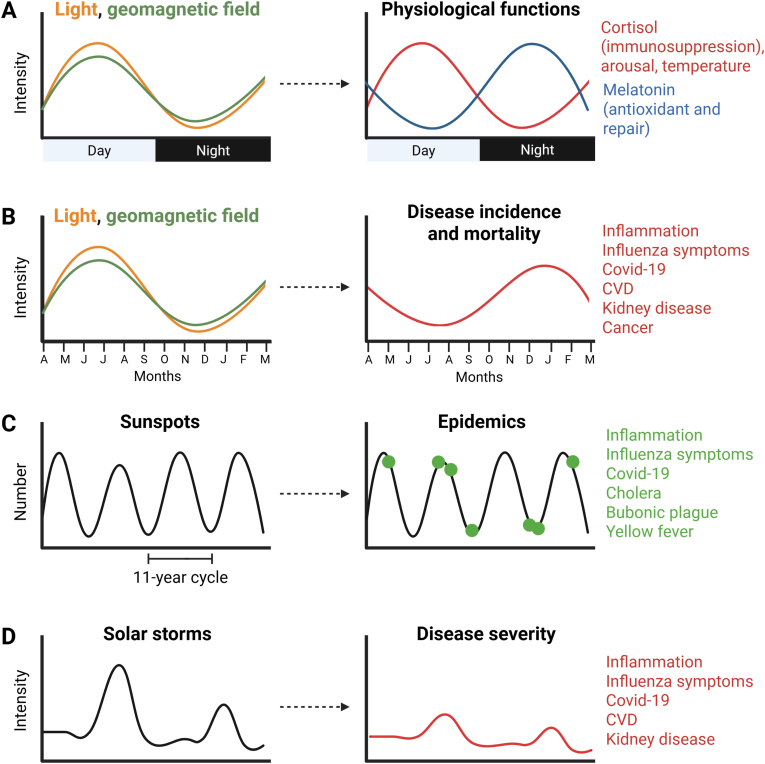
**Solar activity and human health.** Variations in light and intensity of the geomagnetic field according to A) time of the day or B) seasons have been linked with changes in health. In B, conditions for countries at high latitudes in the northern hemisphere are shown as an example. C) Sunspots and D) solar storms may also affect epidemics and disease severity. The graphs are adapted from previous work, including Hope-Simpson [58], Zhou and Stix [59], Martel et al. [34], Nasirpour et al. [65], and Sergeenko [42], and the references cited therein. Only average seasonal geomagnetic field intensity for northern latitudes is shown for simplification. Abbreviation: CVD: cardiovascular disease.

The human brain produces ELF electromagnetic signals that are characteristics of mental activity: alpha brain waves (8–13 Hz) are associated with a calm and relaxed state and closed eyes; beta waves (13–30 Hz) occur during active thinking and reasoning; delta (1–4 Hz) and (4–8 Hz) theta waves are associated with deep and light sleep, respectively. Given the similarities between the first two modes of Schumann resonances and brain waves as well as the possibility of affecting mental reasoning with exogenous delta and theta waves, it was suggested that natural electromagnetic fields entrain circadian and physiological activities by inducing resonance [34,36,54] [Fig. 5]. A series of oscillators can synchronize and entrain to a particular frequency, a phenomenon of resonance that brings coherence to the system, whereas inflammation and disease states may be associated with decoherence and disrupted rhythms. For instance, the circadian pacemaker may facilitate brain activity associated with arousal during wake time, while possible resonances between the brain and peripheral organs can also occur.

Nasal breathing was shown to induce neuronal oscillations and brain waves of animals [89]. These brain waves were abolished following olfactory bulbectomy or when animals breathed through the mouth instead of the nose [89]. Respiratory-entrained brain rhythms may explain some of the health benefits observed for the practice of taping the mouth during sleep to prevent mouth breathing, which improves sleep quality and reduces daytime stress and common colds [90]. While more research is needed to confirm this possibility, the mouth tape also reduces respiratory symptoms, possibly through the enhanced neuronal oscillations and by preventing dehydration and airway irritation [90].

Reciting the Rosary prayer or a mantra induced synchronized cardiovascular rhythms in healthy volunteers [91]. Enhanced heart rate variability (HRV) and baroreflex sensitivity were attributed to slow breathing of six breaths per minute during recitation. The same breathing exercise is used to induce internal coherence and possible resonance between the human heart and the geomagnetic field [92]. This may improve parasympathetic tone and healing.

Oscillations of the geomagnetic field have been detected around the cages of mice during a touch healing intervention developed by William Bengston to treat tumor growth [93]. These oscillations of 1–8 milli-Gauss showed changes in frequencies from 20 to 30 Hz, 7–8 Hz, and finally to less than 1 Hz, before reversing and increasing again. A coupling between the healer's and subjects' electroencephalograms corresponding to the Schumann resonances and their harmonics was also described during these healing sessions [94]. The touch healing method was repeatedly shown not only to reduce tumor size in the mice, but also cure cancer for the rest of their lifespan [95].

## 9 Interference from electromagnetic pollution

**Fig. 6:**
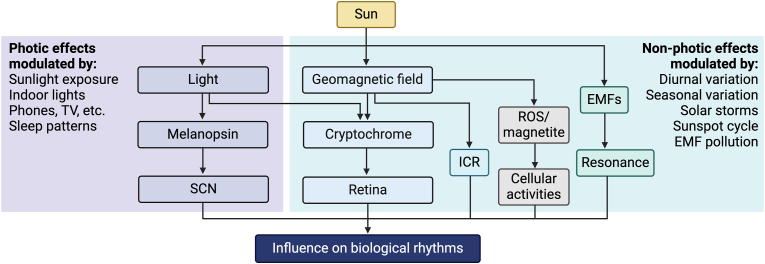
**Effects of light and natural electromagnetic fields on the circadian rhythm and health.** Diagram illustrating the effects of light and native electromagnetic fields on the circadian rhythm. Possible mechanisms and mechanisms of modulation are also indicated. See the text for more detail. Abbreviations: EMFs: electromagnetic fields; ICR: ion cyclotron resonance; SCN: suprachiasmatic nucleus; ROS: reactive oxygen species.

**Fig. 7:**
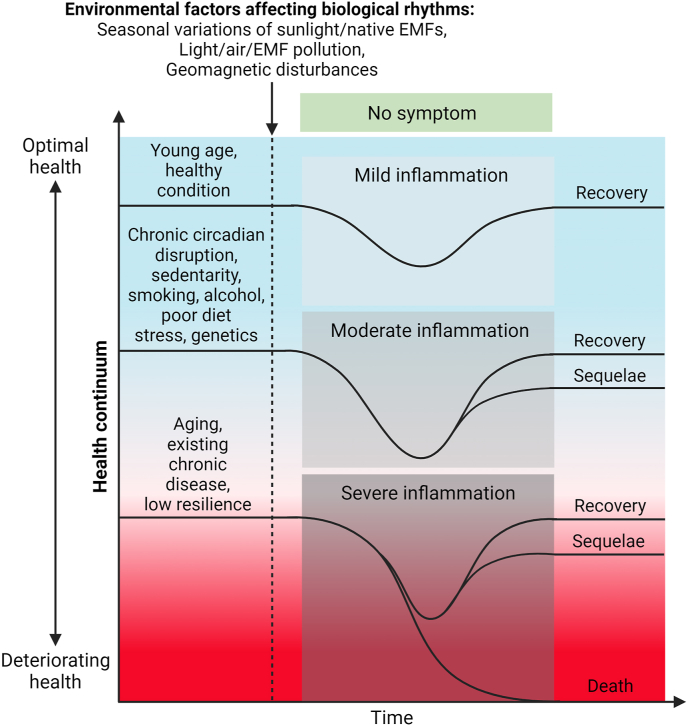
**Factors that affect the circadian rhythm and human health.** Various endogenous and exogenous factors may affect the circadian rhythm. An accumulation of detrimental factors may produce pronounced health effects, especially when combined with aging, pre-existing chronic diseases, and low resistance capacity. Abbreviation: EMF: electromagnetic field.

Weak radiofrequency and microwave radiation used for wireless telecommunications can disrupt magnetoreception and affect a wide range of physiological functions in insects, birds, plants and animals, including growth, development, fertility and behavior [96]. For insects and birds, this form of interference with their magnetic compass may be fatal as they fail to find their route to their favorable habitat. Of note, chronic exposure to man-made ELF magnetic fields, such as those produced by power lines, can reduce melatonin and increase the risk of developing leukemia in children, but only when their intensity is lower than that of the geomagnetic field (<30 μT) [97]. Prolonged and chronic exposure to cell phone and 60-Hz magnetic fields can also reduce melatonin levels in humans [98] [Fig. 6]. Moreover, Wi-Fi electromagnetic radiation reduces the formation of EZ water in vitro [99], suggesting non-specific effects. Weak radiofrequency radiation and pulsed electromagnetic fields may slightly disrupt homeostasis and desynchronize biological rhythms that direct growth, development, metabolism, and body repair. A sudden and massive increase of electromagnetic pollution, such as that produced by new telecommunication systems and the large number of low Earth orbit internet satellites, may affect human health in unexpected ways [66,100], a possibility that warrants further attention and may be analogous to the relevance of widespread and heterogeneous chemical pollutants in the air, food and water supply. Overlapping systems involved in regulation of homeostasis and the circadian rhythm appear to exist in humans and may play a role in the effects of non-ionizing wireless radiation. The health status of the host and other stress factors need to be analyzed when considering the detrimental effects of electromagnetic pollution [Fig. 7]. Disease and symptoms may develop when the cumulative effects of several factors overwhelm the mechanisms of resistance to stress and adaptation, as seen in the elderly or other vulnerable populations. Artificial electromagnetic fields and wireless radiation may also interfere with biological rhythms in an indirect manner by affecting internal coherence, which in turns leads to alterations in rhythmic biological processes. Subtle alterations of coherence by global sources of electromagnetic radiation may be sufficient to increase disease symptoms worldwide.

## 10 Conclusions

We describe a series of observations indicating that light exposure, lifestyle habits, and electromagnetic fields regulate and influence biological rhythms in living organisms and humans. Strategies to improve the circadian rhythm include enhancing exposure to sunlight during the day, phasing-out of blue-enriched LED lights, and reducing blue light in the evening and at night. Exercising during late afternoon and evening may enhance the beneficial effects on health. Better information related to space weather and solar storms and education about the effects of seasons, the solar cycle and circadian disruption on health may help people and especially the elderly to prevent some of the detrimental effects of these environmental energy fluctuations. Furthermore, activities that can induce the formation of liquid crystalline water include regular exposure to sunlight, infrared light, spas, saunas, showers, and grounding in natural environments. The use of a sleep tape at night and slow nasal breathing represent other strategies that can improve internal coherence. Finally, reducing technology that produces electromagnetic pollution and wireless radiation could prevent circadian disruption.

Humans are sensitive to even small variations of light, temperature, and electromagnetic fields in the environment. It might not be entirely surprising that artificial light, solar storms, and sunspots can affect a wide range of disease and symptoms. Thus, the circadian rhythm, sunlight, natural and anthropogenic electromagnetic fields and water may have a larger effect on human health than usually considered.
